# An Internet Access Survey for People With Learning Disabilities in Wandsworth, London

**DOI:** 10.1192/bjo.2025.10678

**Published:** 2025-06-20

**Authors:** Haramrit Sohal, Ayodele Peters, Anna Sri

**Affiliations:** South West London and St George’s Mental Health Trust, London, United Kingdom

## Abstract

**Aims:** Research from the 2000s onward reveals significant digital access disparities for people with learning disabilities (LD). These individuals often have lower rates of computer ownership, internet use, and digital skills compared with their non-disabled peers. The situation is more pronounced for those with co-existing mental health conditions, leaving them further excluded from digital rights. Few studies have explored the reasons behind this digital divide or proposed solutions to improve internet access.

The study was aimed at gaining insight into the internet access and use of social media in patients with a Learning Disability in Wandsworth.

**Methods:** A retrospective cohort study was conducted involving fifty clients from the Psychiatry caseload of Wandsworth Mental Health and Learning Disability Team. The clients, selected randomly, had a range of intellectual disabilities and lived in different settings (e.g. with parents, independent housing with carers, or nursing homes). Data was gathered via an accessible paper survey between August 2024 and February 2025. Doctors filled out the forms in the presence of the clients or their carers to reduce recall bias. The survey collected demographic data and explored internet access, usage, and social media habits. It also included questions on internet safety and barriers to use. Chi-square tests were used to analyse relationships between variables.

**Results:** The study found that a significant number of clients had internet access. Most clients used the internet to watch videos on platforms like YouTube or Google, while some played online games. A smaller number used social media platforms, including Facebook, Instagram, and TikTok. However, many clients had limited understanding on cyber-safety and were unaware of accessibility features for those with visual or hearing impairments. Concerns were raised for a small group of clients who had shared personal or vulnerable information on social media. Thematic analysis identified four main barriers to digital inclusion: lack of access to devices, insufficient support from carers, lack of training, and physical/cognitive challenges.

**Conclusion:** The growing use of social media among individuals with intellectual disabilities highlights the need for targeted internet safety training. Without proper guidance, clients are at risk of online exploitation. In response, a one-day workshop on internet safety was organized with input from speech therapists, psychologists, and IT professionals. Feedback from participants will help assess the effectiveness of the training. The goal is to expand the study to other teams within Southwest London Trust and explore more objective data, such as device usage logs, alongside self-reported information.

